# Intermittency ratio: A metric reflecting short-term temporal variations of transportation noise exposure

**DOI:** 10.1038/jes.2015.56

**Published:** 2015-09-09

**Authors:** Jean Marc Wunderli, Reto Pieren, Manuel Habermacher, Danielle Vienneau, Christian Cajochen, Nicole Probst-Hensch, Martin Röösli, Mark Brink

**Affiliations:** 1 Empa Laboratory for Acoustics/Noise control, Swiss Federal Laboratories for Materials Science and Technology, Duebendorf, Switzerland; 2 n-Sphere AG, Zurich, Switzerland; 3 University of Basel, Basel, Switzerland; 4 Swiss Tropical and Public Health Institute, Basel, Switzerland; 5 Psychiatric Hospital of the University of Basel, Center for Chronobiology, Basel, Switzerland; 6 Federal Office for the Environment, Bern, Switzerland

**Keywords:** annoyance, cardiovascular effects, emergence, fluctuation, intermittent noise, sleep disturbance

## Abstract

Most environmental epidemiology studies model health effects of noise by regressing on acoustic exposure metrics that are based on the concept of average energetic dose over longer time periods (i.e. the *L*_eq_ and related measures). Regarding noise effects on health and wellbeing, average measures often cannot satisfactorily predict annoyance and somatic health effects of noise, particularly sleep disturbances. It has been hypothesized that effects of noise can be better explained when also considering the variation of the level over time and the frequency distribution of event-related acoustic measures, such as for example, the maximum sound pressure level. However, it is unclear how this is best parametrized in a metric that is not correlated with the *L*_eq_, but takes into account the frequency distribution of events and their emergence from background. In this paper, a calculation method is presented that produces a metric which reflects the intermittency of road, rail and aircraft noise exposure situations. The metric termed intermittency ratio (*IR*) expresses the proportion of the acoustical energy contribution in the total energetic dose that is created by individual noise events above a certain threshold. To calculate the metric, it is shown how to estimate the distribution of maximum pass-by levels from information on geometry (distance and angle), traffic flow (number and speed) and single-event pass-by levels per vehicle category. On the basis of noise maps that simultaneously visualize *L*_*eq*_, as well as *IR*, the differences of both metrics are discussed.

## Introduction

Transportation noise, largely from road, railway and aircraft traffic, is one of the most widespread sources of environmental stress and discomfort in daily life. Health effects of noise may emerge directly via autonomous stress reactions to the physical exposure or indirectly via negative affective states, for example, annoyance. Noise pollution is an important public health factor, with recent burden of disease estimates ranking it the second major environmental health risk after air pollution in Europe.^1^ A recent review^2^ summarized the current state of knowledge about auditory and non-auditory effects of noise.

### Health Effects of Transportation Noise

The most prevalent non-auditory effect in a population exposed to environmental noise is annoyance. It may result from interference with daily activities, rest or sleep, and can be accompanied by negative emotional and behavioral responses such as anger, displeasure, exhaustion and by stress-related symptoms.^3, 4^ Importantly, humans perceive, evaluate and react to environmental sounds not only during daytime, but also when asleep. Laboratory and field studies have consistently demonstrated that transportation noise induces acute and short-term effects on sleep.^5, 6^ Acute physiological reactions to noise events during the night include conscious and unconscious awakenings, shifts to lighter sleep stages, cortical and cardiovascular arousals (increases of heart rate and blood pressure) and body movements.^6, 7^ The probabilities of such reactions are clearly correlated with acoustic characteristics of noise events, especially with the maximum sound pressure level and the slope of rise of the level. In field studies, noise events with *L*_Smax_ as low as about 35 dB(A) at the ear of the sleeper have been shown to induce electroencephalography awakening reactions in sleeping environments with low background noise.^8, 9, 10^ Recent evidence as, for example, from the HYENA study^11^ suggests that nocturnal noise exposure may be more relevant for the genesis of long-term cardiovascular outcomes than daytime noise exposure, probably because of repeated autonomic arousals (short-living heart rate and blood pressure increases, peripheral vasoconstriction and so on) during sleep that have been shown to habituate to a much lesser degree than cortical arousals.^5, 12^ A recent Swiss study presented evidence of an adverse effect of railway noise on blood pressure, that was especially associated with night-time exposure.^13^ As it is well plausible that an ample proportion of the long-term cardiovascular health impacts of noise are triggered by repeatedly occurring cardiovascular arousals during sleep,^14^ it is paramount for a (new) noise metric to amply reflect the acoustic characteristics of the noise events that potentially trigger such arousals.

### Differences between Constant and Intermittent Noise

Many epidemiological studies, as well as many annoyance surveys consider noise exposure as equivalent continuous levels over longer time periods (e.g., *L*_dn_, *L*_den_, *L*_night_ or *L*_day_^15, 16^). It has been repeatedly shown that such energy-based exposure measures (alone) have limited explanatory power regarding annoyance or disturbance effects—yet essentially better and more sophisticated acoustic metrics to explain noise effects are likewise missing. This has earned the *L*_eq_ a reputation of being the "best of all the bad noise exposure metrics". However, the application of equivalent levels to predict the impact of noise on sleep has not met with much success.^17^ Whereas the probabilities of event-related awakenings and cardiovascular arousals clearly increase with the maximum sound pressure level of noise events, average noise metrics usually fail to predict noise-induced sleep disturbances sufficiently.^6, 18, 19^ One can conclude that depending on whether the noise source is intermittent (such as passing flights or trains) or continuous (such as road traffic from a busy highway), the effects of noise on sleep might be better explained by the number of noise events and their characteristics, than by average noise exposure.^8^

**Figure 1:**
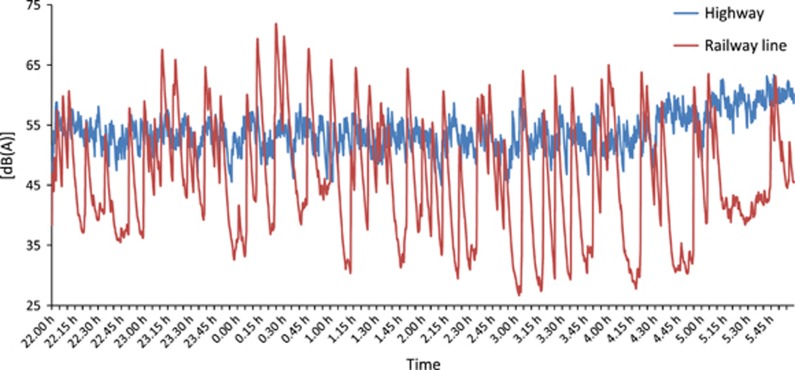
Level-time course of the sound pressure level produced by road traffic on a highway, at 7.5 m distance, and along a railway line with predominantly freight traffic events, at 250 m distance, for a time period of 8 h. Both signals have been recorded along the Swiss north-south transit axis on the Gotthard route (road and rail) between 22 and 06 h and normalized to 55 dB(A) *L*_eq_.

Figure 1 illustrates the effect of different degrees of source intermittency on the course of the sound pressure level for a given average exposure level (*L*_*eq*_=55 dB(A)) over an 8-h observation period, recorded along a railway double-track line (red) and a highway (blue). Although, in this example, both exposure situations yield the same average level during the observation period, the higher degree of intermittency of individual events, which is characteristic to railway traffic, produces more strongly pronounced fluctuations in the sound pressure level.

It is highly questionable that the two exposure situations shown in Figure 1 result in the same overall effect during any given time period. Although a larger impact on sleep by the exposure from the railway line at night time can be expected for the above mentioned reasons, the presence of calm periods of respite in between loud pass-by events may render the same railway noise more acceptable during daytime. The latter is reflected in the "rail bonus" which is inherent to many railway noise regulations in Europe. The constant noise from the highway on the other hand might pose no threat to a good night's sleep, while being considerably annoying at daytime. It is thus well possible that the established source dependence of reported annoyance (in particular, as reviewed by Miedema et al.^20^) can at least partially be explained by differences in noise variation over time.

It is important to note that the temporal variation characteristics of noise do not just vary between different source categories (road, rail and air), but also within the same source. This is most clearly obvious in road traffic noise, where the temporal structure of occurring noise events shows a high variation between small one-lane city streets with highly intermittent noise up to wide 4-, 6- or even 8-lane highways, which produce a nearly continuous sound exposure with very little fluctuation. Miedema concluded that highway noise produced higher annoyance as compared with main roads at comparable *L*_Aeq24h_.^21^ In contrast Lercher et al.^22^ found higher annoyance prevalences for main-road traffic than for highway traffic noise of the same *L*_den_ level. The same group also found that the distance between dwellings and the nearest railway track was a level-independent predictor for railway noise annoyance, with higher annoyance ratings in people living <300 m from the railway track.^23^ These findings suggest an influence of the temporal structure of noise exposure on annoyance reactions—and maybe other health outcomes as well—however, the functional relationship is yet unclear.

### Metrics for Acoustically Characterizing Intermittent Noise Situations

No matter how one looks at it, it seems essential that large-scale long-term health effect studies, as well as annoyance surveys, should consider more detailed characteristics of the temporal variation of the sound, as well as its emergence instead of only considering averaged exposure levels. In the last decades there have in fact been several proposals to add time-related variables to replace or supplement the *L*_*eq*_. A discussion of alternatives or amendments to the *L*_*eq*_ can be found in TSG9^24^ and Commission E.^25^ Common approaches are the introduction of thresholds and the counting of the number and duration of events (e.g., the Noise and Number Index, which was common during the 1970s and 1980s) or an application of level statistics. The latter are, for example, the basis of derived quantities such as the Traffic Noise Index,^26^ the Noise Pollution Level,^27^ the Common Noise Index developed within the Harmonica project,^28^ the concept of notice-events ^29^ or fluctuation and emergence, as used by Bockstael et al.^30^ However, these metrics have so far not reached a broader application for regulatory purposes either because they highly correlate with the *L*_*eq*_ or for reasons of complexity to be implemented in or combined with common calculation models.

### Rationale and Goals

Although the association between (night time) noise and health outcomes could be convincingly demonstrated in past research, the causal chain from reactions to individual noise events in the night to the long-term outcomes is not yet fully understood. We hypothesize that for a given exposure level the potential of noise to activate pathophysiologically relevant pathways is more pronounced in noise situations that produce relatively few but loud single events with calm periods in between, than constantly emitting noise sources with almost no variation of the sound pressure level over time. If this would hold true, one should observe higher risk estimates for cardiovascular and other health outcomes in exposure situations that emit noise intermittently rather than constantly. Therefore, a measure of intermittency may contribute, at least in part, to elucidate the large proportion of unexplained variance which is usually found in noise effect models that predict effects from average exposure solely. Such a measure could therefore be used as a second predictor metric, complementing energy equivalent average level metrics such as the *L*_*eq*_.

The present work was stimulated by the demands of the *SiRENE* study^31^ which investigates transportation noise effects in the Swiss population. One goal of the project is to elucidate the effect of source intermittence on cardiovascular morbidity and mortality in two large-scale epidemiological studies (the *SAPALDIA* cohort and the *SNC* study). We aimed at deriving an exposure metric to quantify the intermittence and put to test the working hypothesis mentioned above. In this paper we thus present a calculation method by which current noise exposure models can be extended with a metric that yields an integral description of the eventfulness (or intermittency) of noise exposure situations, taking into account both number and magnitude of noise events during a certain time period. The metric referred to as intermittency ratio (*IR*) can be derived either directly from acoustic measurements or calculated from traffic and geometry data for any transportation noise source and any time period (including day and night).

## Methods

In the next sections, a calculation method is described by which *IR* can be integrated in current noise exposure calculation models. The method has been implemented in *sonBASE*, the Swiss noise mapping database^32^ and *IR* has been calculated for entire Switzerland. The result of this effort is presented in the Results section where noise maps are shown that incorporate the *IR* metric alongside *L*_*eq*_.

### *IR* Basic Principle

Highly intermittent traffic noise exposure situations consist of subsequent pass-bys of vehicles (cars, aircraft, trains and so on) which acoustically stand out from the background (noise) by a certain degree. We define such parts of the level-time course as "noise events". A noise event can be characterized by its maximum level, its sound exposure level, the emergence from background noise, its duration, or by the slope of rise of the level. For an integral characterization of the "eventfulness" of an exposure situation over a longer period of time we introduce the event-based sound pressure level *L*_eq,T,Events_, which accounts for all sound energy contributions that exceed a given threshold, that is, clearly stand out from background noise. This event-based sound pressure level *L*_eq,T,Events_ can now be compared with the overall sound pressure level *L*_eq,T,tot_. The *IR* is defined as the ratio of the event-based sound energy to the overall sound energy.

Hence *IR* is defined as


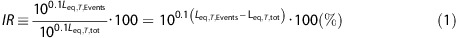


*L*_eq,T,tot_ corresponds to the equivalent continuous sound pressure level of all sound sources involved and is given as


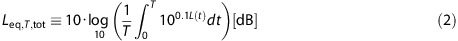


where *L*(*t*) is the continuous sound pressure level at the receiver position.

A single pass-by only contributes to *L*_eq,T,Events_ if its level exceeds a given threshold *K*.


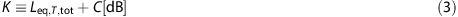


This threshold *K* is defined relative to the long-term average of the overall sound pressure level *L*_eq,T,tot_ and an offset *C*. Thus *L*_eq,T,Events_ is defined as:


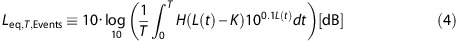


with the Heaviside step function *H*. The offset *C* is the only free parameter within the definition of *IR*. On the basis of practical experience on transportation noise situations, *C* might not be smaller than 0 and not larger than about 10 dB. For low values of *C*, almost any situation produces a large *IR*, whereas high values of *C* almost always produce low *IR*, as only in extraordinarily intermittent situations the level rises above the high threshold. This means that such choices of *C* cause greatly skewed distributions of *IR*. To be able for *IR* to distinguish between situations with different degrees of intermittency, the criterion for setting *C* was a preferably uniform spread of *IR* across the range of exposure situations as they occur in the real world. The balance between these extreme cases was investigated by numerical simulations of various traffic situations and resulted in *C*=3 dB.

The integration time *T* has to be chosen in a way that the partial *L*_*eq*_s do not significantly differ from the total *L*_*eq*_. If this precondition is not fulfilled, for example, because of a highly varying traffic flow, it is recommended to determine partial Intermittency Rates and average them according to the equation given in Appendix section A7.

**Figure 2:**
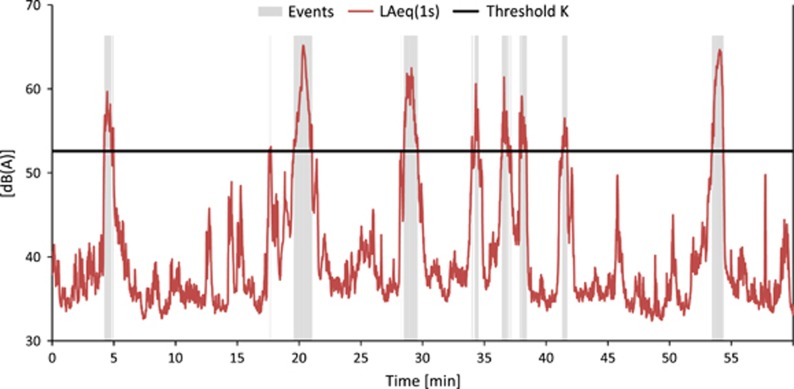
Railway noise recordings at 560 m distance from a freight train railway line during the night. Background noise is dominated by nature sounds and distant road traffic noise. Intermittency ratio (*IR*)=87%. The portions of the curve marked “gray” are used to calculate *L*_eq,T,Events_.

**Figure 3:**
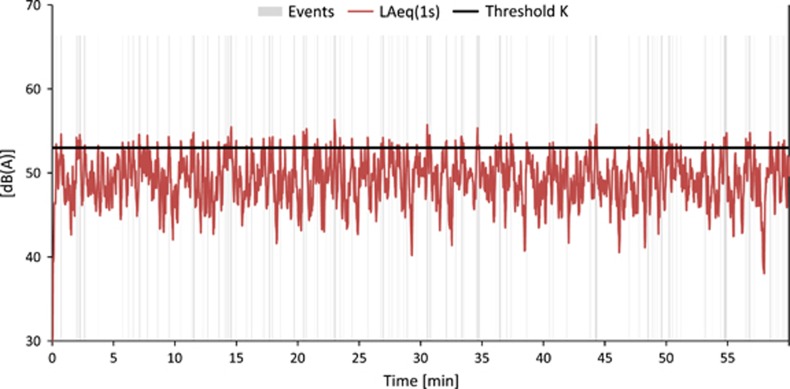
Road traffic noise at 100 m distance from a highway with a speed limit of 120 km/h. A total of 3200 vehicles per hour with 12% heavy traffic. Intermittency ratio (*IR*)=19%. The portions of the curve marked “gray” are used to calculate *L*_eq,T,Events_.

By definition, *IR* only takes values between 0 and 100% (including 0% and 100%). An *IR* of >50% means that more than half of the sound dose is caused by "distinct" pass-by events. In situations with only events that clearly emerge from background noise (e.g., a receiver point close by a railway track), *IR* yields values close to 100%. As, for example, Figure 2 shows a railway noise situation where the single events exhibit FAST-weighted maximum levels which are about 20 dB(A) higher than background noise and a high *IR* of 87%. In contrast Figure 3 depicts a situation with intensive road traffic at 100 m distance. Here, the levels are rather constant over time and consequently, the *IR* is low with only 12%.

**Figure 4:**
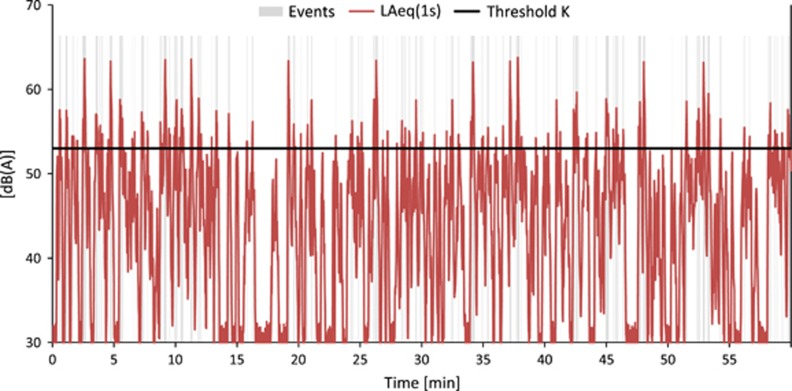
Road traffic noise at 100 m distance from a highway with a speed limit of 120 km/h. A total of 315 vehicles per hour with 12% heavy traffic. Intermittency ratio (*IR*)=62%. The portions of the curve marked “gray” are used to calculate *L*_eq,T,Events_.

Air and railway traffic generally exhibit a high *IR*, with the exception of situations with such a high background noise (e.g., noise from other sources) that the events are partially or fully masked. The *IR* for road traffic is primarily determined by the number of pass-by events and by the distance between source and receiver. Figure 4 shows another road traffic situation with the same overall *L*_*eq*_ as Figure 3 but a shorter distance to the source and less vehicles per hour, resulting in a significantly higher *IR* of 62%.

### Estimation of the *IR* by Calculation

**Figure 5:**
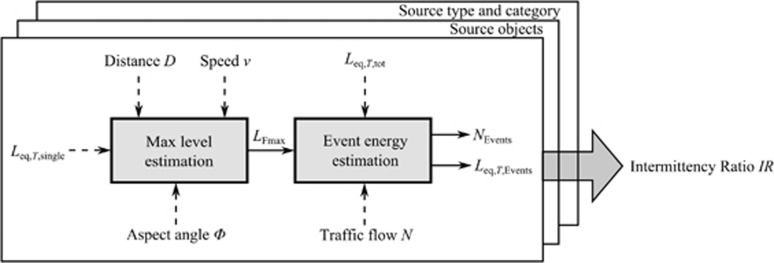
Scheme for the calculation of the intermittency ratio (*IR*).

In the examples shown above, the IR was derived based on measured level *vs* time data. However, with only a few exceptions (such as, for example, the aircraft noise model FLULA2^33^, transportation noise calculation models that can be used for large-scale calculations such as CNOSSOS,^34^ Harmonoise,^35^ Nord2000^36^ or Doc. 29^37^ are designed in a way that only permits equivalent continuous sound pressure levels *L*_eq_ or derived quantities such as *L*_den_ as an output, but no information on level variation over time. Therefore, a direct calculation of the IR is not possible in these models. However *IR* can be estimated based on traffic information which is commonly at hand for noise assessment purposes. The corresponding procedure is illustrated in Figure 5.

The first step is to assess the maximum sound pressure level *L*_Fmax_ of single pass-by events. The estimation of maximum levels as given in equation (5) is based on the assumption that the propagation conditions for the entire source are also representative for the geometry that determines the maximum level. This might not hold in the case of strong changes of shielding effects, that is, if barriers abruptly end along the line source. Therefore in such situations it is recommended to split the source into different sections with uniform propagation conditions.

The maximum level of a single pass-by in a given source–receiver geometry can be written as


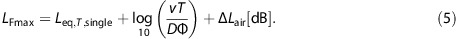


Where *D* is the shortest distance of the source polygon to the receiver point, *v* denotes the speed, Φ is the source path aspect angle, that is, the angle that the source path subtends at the receiver, and Δ*L*_air_ denotes a correction for air absorption. A derivation of equation (5) can be found in Appendix section A1. Equation (5) is valid for point sources. In Appendix section A2 an expression for the maximum level of moving line sources with finite size is given, which should be used in case of trains.

As a next step, for each vehicle category a distribution of maximum levels is derived by adding a source level variance. It is assumed that the maximum levels of single pass-bys are normally distributed. For road traffic an additional variation in level is introduced to account for the temporary overlap of single pass-by events (see Appendix section A3 for details).

**Figure 6:**
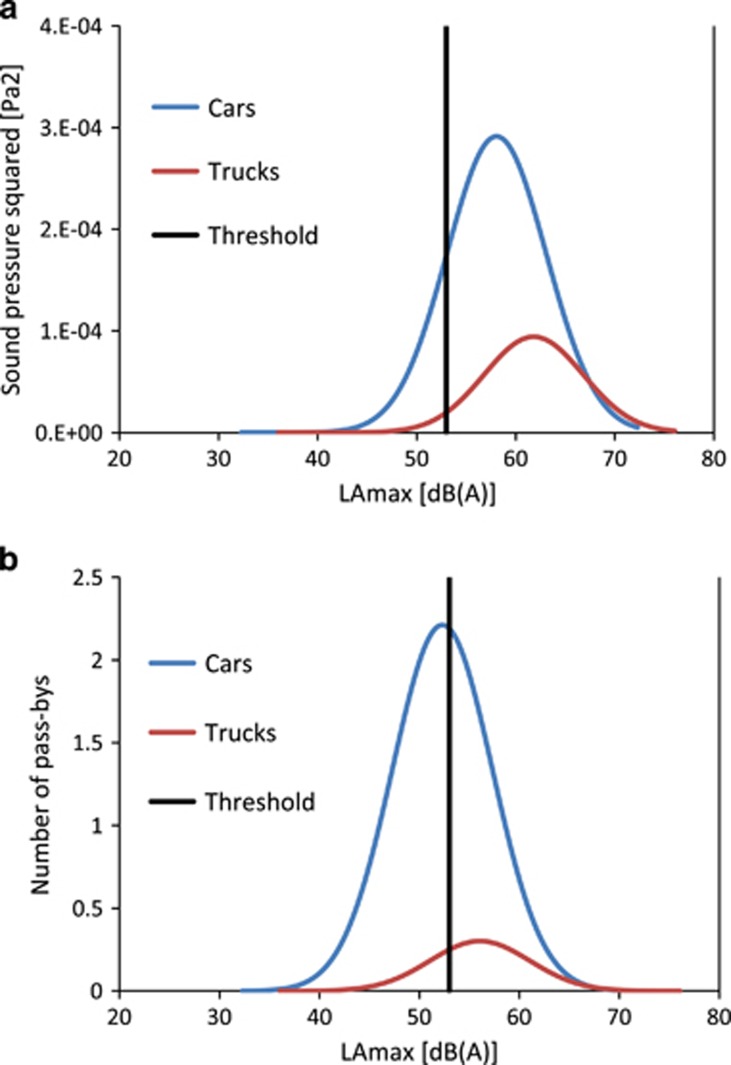
Calculated maximum level distributions for the traffic situation presented in Figure 4, **a**: sound pressure squared, **b**: number of events. Calculation details: 277 cars with 120 km/h and a single-event *L*_eq_ of 24.0 dB(A) and 38 trucks with 90 km/h and a single-event *L*_eq_ of 29.0 dB(A). *L*_eq,T,tot=_50.0 dB(A), *L*_eq,Events=_47.8 dB(A), *IR=*60%, *N*_Events_=151. Resolution of the *x*-axis: 0.1 dB.

As an example, Figure 6 shows calculated maximum level distributions for the traffic situation presented in Figure 4. In Figure 6a sound pressure squared of the maximum pass-by levels, and in Figure 6b the number of pass-by events is shown. The vertical black line indicates the threshold *K*. The number of pass-bys *N* above threshold is calculated according to Appendix section A4. *N* represents an estimate of the number of events. As can be seen, the majority of trucks produce maximum levels above the threshold, that is, noise events, but less than half of the passenger cars do so.

Looking at Figure 6a depicting sound intensity, one would assume a much higher *IR* than 60% as only a small part of the level distribution remains below the threshold. However, it has to be considered that only maximum levels are shown. Even for single pass-bys with maximum levels clearly above *K*, a significant part of *L*(*t*) remains below the threshold. This aspect is taken into account by introducing a weighting function as defined in Appendix section A6 that cuts off that part of an event which remains below the threshold.

As a last step, according to equation (1), *IR* is calculated for all traffic noise sources individually, as well as for the sum of them.

## Results

### Including the *IR* in Noise Mapping

**Figure 7:**
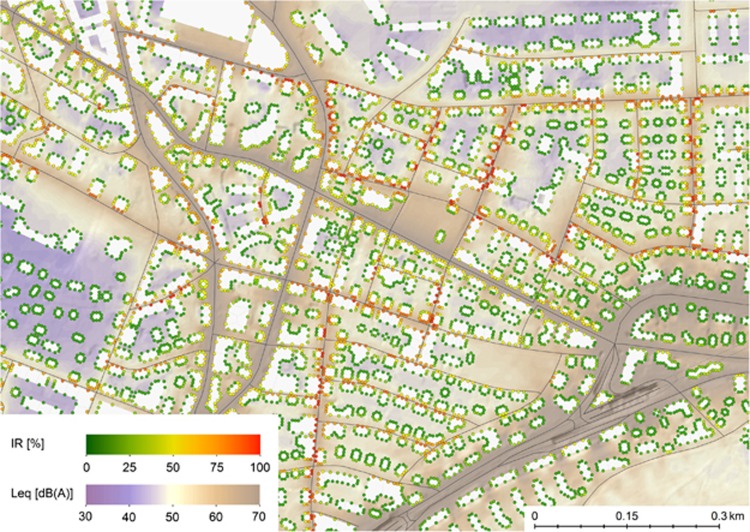
Road traffic noise map showing both *L*_eq_ and *IR* during daytime (16 h). Intermittency ratio (*IR*) has been calculated for individual facade points on the map, marked as small dots. The map section shows an area north of the city of Zurich in vicinity of Zurich airport.

**Figure 8:**
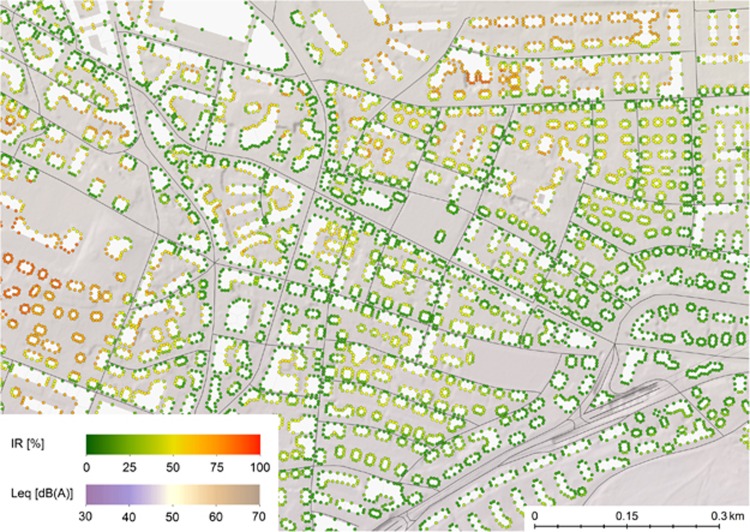
Aircraft noise map showing both *L*_eq_ and *IR* during daytime (16 h). Intermittency ratio (*IR*) has been calculated for individual facade points on the map, marked as small dots. The map section shows an area north of the city of Zurich in vicinity of Zurich airport.

As part of the *SiRENE* study, *IR* was calculated for all dwellings in Switzerland (54,300,000 facade points, assigned to 1,813,000 buildings). As an example, Figures 7 and 8 depict noise maps that incorporate *IR* for an area north of the city of Zurich. Figure 7 shows the road traffic exposure situation during the day. The background color gradient of the map indicates the yearly average for road traffic noise *L*_day_ ranging from 30 to 70 dB(A). As can be seen, important noise sources are a highway/expressway that passes the map section in the lower right corner, and several major roads. In addition, there are numerous arterial roads with significantly less traffic. Values for *IR* have been calculated at all facade points. The facade points are shown as dots, reflecting the value of *IR* with different colors. Low values of *IR* can not only be found far away from roads but also in vicinity of the highway, despite the very different *L*_*eq*_s. In contrast, high values of *IR* can be found close to smaller roads with thinned-out traffic where single pass-bys of vehicles clearly stand out from background noise. Generally, receiver points on the front-side of buildings exhibit a markedly higher *IR* than on the back-side, where noise is much more diffuse.

The influence of background noise on *IR* can be studied well in Figure 8. The same map section as above is used, but the image shows aircraft noise exposure. Disregarding background noise, aircraft noise is basically always "eventful", however, in presence of an elevated background noise (e.g., through road traffic), aircraft flyover events can be masked, resulting in a reduction of *IR*. In the map section shown in Figure 8, the 16-h-*L*_*eq*_ produced by aircraft is almost identical everywhere on the map, at a comparably low level of about 47 dB(A). At receiver positions with elevated road traffic noise, aircraft noise events only cause small *IR*. Elevated levels of *IR* for aircraft noise can only be found far away from major roads where the overall road traffic *L*_*eq*_ is low, such as for example, on the left side and on the right upper corner of Figure 8.

### Correlation between *L*_*eq*_ and *IR*

As most of the common noise metrics like *L*_day_, *L*_den_, or *L*_dn_, to name just a few, are usually highly correlated (e.g., for road traffic noise, *r* is clearly >0.9), the introduction of a complementary (new) metric only makes sense if it is basically uncorrelated with the *L*_*eq*_, as was, for example, pointed out by the authors of the I-INCE report on supplemental noise metrics.^24^ Table 1 lists the correlations between different conventional noise metrics and *IR* for the map section shown in Figures 7 and 8.

Compared with correlations between the most common metrics, the correlations between the *L*_*eq*_ and *IR* in the map section above are rather small. One could expect the correlation to be positive though, for quite obvious reasons. For example, facade points pointing towards a road are characterised by a markedly higher *L*_*eq*_ as those pointing to a rather calm backyard, where the distance to passing vehicles is longer and hence, both *L*_*eq*_ and *IR* are smaller. For aircraft noise, the *L*_*eq*_ during day is very similar for all facade points within the map section, but due to different masking effects from other sources, aircraft *IR* values nearly spawn the whole possible range of values, thus only correlating little with the *L*_*eq*_.

## Discussion

Typically, studies on health and annoyance effects of noise rely on acoustic exposure metrics that are based on the concept of an average energetic dose over longer time periods (e.g., *L*_*eq,24h*_, *L*_dn_, *L*_den_, *L*_night_ or *L*_day_). However there are strong indications that these *L*_*eq*_-based quantities are not appropriate to predict noise-induced sleep disturbances and also have their limitations with respect to satisfactorily explaining variance of annoyance or disturbance of activities. Potentially, other metrics can add to, improve, or replace the predictions made using *L*_*eq*_-based measures. Therefore in this paper, we developed the metric *IR*. This metric allows an integral characterization of the eventfulness of a noise exposure situation, independent of the overall energetic dose. The decorrelation from the *L*_*eq*_ is an eminent feature of a complimentary noise metric that aims at explaining remaining variance in epidemiological studies, and in a practical example, we could demonstrate that the *IR* metric is essentially uncorrelated with energetic measures.

An advantage of the concept of *IR* is that it can be derived based on acoustic measurements, as well as, from calculations as it has been designed in a way which facilitates subsequent implementation into the most common traffic noise calculation models. The latter allows it to be used in large-scale epidemiological (population-based) studies and other types of observational investigations such as annoyance surveys. However, one has to be aware of the fact that in the procedures described, the estimation of maximum levels is based on several assumptions. An increase of the accuracy of the calculation of *IR* could be achieved by applying calculation models that directly yield maximum levels of single pass-bys—or even better—entire level *vs* time courses. First approaches for such models are at hand (for example see de Coensel et al.).^38^

As part of the calculation of *IR*, the decision whether an "event" accounts for *L*_eq,T,Events_ (according to equation (4)) or not, is based on a simple criterion, the parameter *C*, for which a value of 3 dB has been assumed. This value has not been set based on any verified psychoacoustic principle but was derived empirically to attain a uniform spread of *IR* across the range of the different exposure situations. The question of how much an event really has to stand out from background noise in order to be termed "event" by normal listeners depends on various other parameters (which were not addressed in the present paper). In fact, for the noticeability of an event, not only the acoustic characteristics of the event compared to the background, but also the attentional, cognitive and emotional situation of the listener is relevant, as was described by de Coensel et al.^29^

In the near future, we expect to be able to increase the explanatory power of our epidemiological models by incorporating *IR* as a predictor alongside with average level measures within the aforementioned *SiRENE* study. The *IR* metric with its current parameter setting of *C* will be further validated using a self-report rating scale that measures the subjective perception of intermittency by residents affected by noise. In order to further investigate if *IR* is an appropriate measure to describe the relevant aspects of the temporal pattern of noise exposure, we would like to encourage others to implement the methodology presented here in their own models.

## Figures and Tables

**Table 1:** Pearson correlation coefficients (rounded to two decimals) between *L*_eq_- and *IR*-based measures at facade points from the map section shown in Figures 7 and 8. (*N*=35,704 facade points).

	*L_day, air_*	*L_night, air_*	*L_day, rail_*	*L_night, rail_*	*L_day, road_*	*L_night, road_*
*IR* day, air	0.28					
*IR* night, air		0.34				
*IR* day, rail			0.10			
*IR* night, rail				0.23		
*IR* day, road					0.46	
*IR* night, road						0.44

Abbreviation: IR, intermittancy ratio.

## A. Appendix

### A.1 Estimation of the maximum level of a moving point source with constant speed

Taking geometrical spreading and air absorption into account, the effective value of sound pressure, *p*_eff_, of a non-moving monopole point source at distance *r* can be given as

with *W* the sound power, *ρ*_0_ the density of air, *c*_0_ the speed of sound in air and *α* the atmospheric attenuation coefficient in dB/m.

If the point source is moving with constant speed *v* << *c* in m/s along a straight path at distance *D* from a receiver, the effective sound pressure can approximated by

with *t*_*p*_ being the time when the maximum level is reached. Note that *t*_*p*_ − *D*/*c*_0_ corresponds to the time when the source is at shortest distance to the receiver. The FAST-weighted maximum level *L*_Fmax_ is approximated by

Applying the FAST-weighting to a signal emitted by a moving source, leads to a systematic underestimation of its actual level. The magnitude of underestimation depends on the speed *v* and extent of the source *L* as well as the shortest distance to the receiver *D*. In a highway situation with 120 km/h and a measurement distance of 7.5 m relative to the center of the lane the error amounts to approximately 0.5 dB for passenger cars.^39^ For increasing distances or lower speeds the error decreases rapidly. Therefore we decided to neglect that effect in equation (8).

The equivalent continuous sound pressure level *L*_*eq*_ for an integration interval of *t*_*p*_ − *τ* to *t*_*p*_ + *τ* can be given as

by substituting  it can be simplified as

with Φ as the angle that the source path subtends at the receiver. For a pass-by of infinte length Φ = *π*. By neglecting air absorption (*α* = 0) follows

Subtracting (9) from (12) an expression for the maximum level of a constantly moving point source in dependence of the *L*_eq_ can be found

where

Subtracting equation (11) from equation (9) it can be shown that the influence of air absorption on Δ*L* is independent from the speed *v*, but dependent on the distance and the source aspect angle Φ. Assuming a pass-by of infinite length for the influence of air absorption, Δ*L* can be approximated as

Δ*L*_air_ can be given as

Δ*L*_air_ is always positive and increases with increasing air absorption *αD*. For *αD* = 1 dB equation (16) yields Δ*L*_air_ = 1 dB, with *αD* = 3 dB follows Δ*L*_air_ = 2 dB.

By substituting  and hence adapting the integration limits the above expression can be efficiently numerically solved

Applying a Gauss-Legendre integration of third order (*n* = 3)

with *λ* =−0.1*αD*, the error remains below 0.1 dB for *αD* < 7 dB.

Although the atmospheric attenuation coefficient *α* is highly frequency dependent, in order to keep the computational cost low, a representative value per noise source type is proposed. Based on the analysis of representative source spectra it is recommended to set *α* = 5 dB/km for road and air traffic.

### A.2 Estimation of the maximum level of a moving line source with constant speed

The estimation for single pass-by *L*_Fmax_ as given in Appendix section A1 is based on the assumption of a moving point source, which can be used for cars and planes, but not for trains. In this section an extension is presented to derive *L*_Fmax_ for moving line sources of finite length *L*. It is assumed that the sound power *W* is equally distributed over an incoherently radiating line source.

The maximum level is reached for the geometrical situation in which the center of the line source is at shortest distance *D* to the receiver. Integrating over the length of the line source, the sound pressure can be given as

Neglecting air absorption (*α* = 0) it can be deduced

Applying the substitution *s*/*D* = tan *θ* it follows

with  as the opening angle of the source. The maximum level of a moving line source can then be given as

Neglecting air absorption the *L*_eq_ of a single pass-by remains as for point sources, see equation (12). Δ*L* is derived by subtracting (12) from (22):

with

Thereby Φ >> Θ, meaning that the whole source element, i.e., for example the entire railway track, must be clearly longer than the moving line source, i.e., the train. For *L*≪2*D*, Θ can be approximated by , in which case equation (23) takes on the behavior of a point source according to equation (15).

For moving line sources the influence of air absorption is generally slightly smaller than for point sources. Nevertheless the expression for Δ*L*_air_ given in equation (18) can be used as first approximation.

As railway noise sources generally exhibit more high frequent components than road and air traffic it is recommended to set *α* = 7 dB/km for railway traffic.

### A.3 Maximum level distribution

It is assumed that the maximum levels of single pass-bys are normally distributed for a given vehicle category and only slightly varying speed.

with the standard deviation *σ* in dB and the arithmetic mean . However, sound levels obtained from noise calculations usually represent an energetic mean (here denoted *L*^*E*^) over a certain time. Given the above assumption, the following relation between the arithmetic and the energetic mean can be deduced

In real situations, the sound pressure signals of single pass-bys overlap temporarily. By the temporal overlap, the total *L*_eq_, *L*_eq,T,tot_, and—by definition—the threshold *K* are not affected. However, the intensity above the threshold (*L*_eq,Events_) increases and thus the Intermittence Ratio. The influence of the overlap effect on *IR* in the case of road traffic noise was investigated by numerical simulations. Measurement data of periodically and stochastically distributed pass-by times were synthesized as the two marginal cases. It could be shown that particularly intermittency rates between 0 and 0.5 are affected. It was found that this effect could be approximated by an artificial increase of the standard deviation of the level by

with *σ*_0_ being the single source standard deviation and *σ*_OL_ an additional standard deviation for the temporal overlap. At maximal overlap the simulations yielded *σ*_OL_ ≈ 4.5 dB, at minimal overlap however *σ*_OL_ ≈ 2 dB. As a compromise between both marginal cases *σ*_OL_ = 3 dB is adopted for road traffic noise. For railway and air traffic noise the temporal overlap is neglected, i.e. *σ*_OL_ = 0 dB.

The single source standard deviation reflects the variation of the sound power level within a vehicle category at a given speed. For the current application of *IR* within the project *SiRENE* it was concluded that a representative standard deviation *σ*_0_ = 2 dB can be used for all vehicle categories such as passenger cars, heavy and light trucks, different types of trains and aircraft and that the assumption of a normal distribution was valid. For road noise the above conclusions were taken based on measurement data of road traffic monitoring stations collected in the project *Footprint*^40, 41, 42, 43^ based on a classification system with 10 categories (Swiss10). For railway noise measurement data from the project *sonRAIL* was analysed^44, 45^ and for aircraft noise we relied on the emission database of *FLULA2*^33, 46^ and measurements from the *sonAIR* project.^47, 48^ However it has to be kept in mind that this setting is depending on the local (national) composition of the vehicle fleet and its categorization and does not account for significant variations in the travelling speed such as congestions. For other countries a wider spread might be appropriate, see for example.^49^ Therefore the setting of these standard deviations has to be checked and if necessary adjusted to the local conditions.

### A.4 Number of events

The number of events exceeding the threshold *K* can be calculated by

with *N* the traffic flow intensity, i.e. the number of pass-bys within time *T*, and with the Gauss error function erf().

### A.5 Event-*Leq*

The Event-*Leq L*_eq,T,Events_ is calculated by

where *w* denotes a weighting function and

Equation (29) has to be evaluated for different source types (regarding the parameters *L*_eq,T,single_, *D*, *v*, Φ) separately. On the basis of the partial Event-*Leq*s *L*_eq,T,Events,i_ the total *IR* is calculated by

In doing so, the temporal overlap between different source types is neglected which underestimates the *IR*. As pass-bys of aircraft and trains generally only last a small percentage of time this effect is of minor importance.

### A.6 Weighting function

The weighting function *w*(*x*) considers the truncation effect due to the finite line source length and makes sure that for a given event only the intensity above the threshold *K* is integrated. *w*(*x*) is formulated as

with *x* = *L*_Fmax_ − *K*. *f* corresponds to the analytical solution of a line source neglecting air absorption and *g* is an empiric correction for the influence of the air absorption. It can be shown that

The influence of the air absorption is approximated under the assumption of an infinitely extended source (i.e. Φ = *π*). Investigations by numerical simulations yielded the empirical model

with parameters *b* and *c* for which the following linear regressions were obtained

### A.7 Averaging of intermittency rates over different time periods

Averaging of *IR* over *M* time periods *k* is defined as

The Intermittency Ratio *IR* of time period *k* is weighted with the total *Leq* during that period. Hence time periods with higher *Leq* get a higher weight on *IR* than periods with lower *Leq*.

The averaging has to be performed as the final step, after the summation of *IR* over different sound sources as defined in Appendix section A5.
